# Aberrant central plasticity underlying synchronous sensory phenomena in brachial plexus injuries after contralateral cervical seventh nerve transfer

**DOI:** 10.1002/brb3.2064

**Published:** 2021-02-06

**Authors:** Zeyu Cai, Gaowei Lei, Jie Li, Yundong Shen, Yudong Gu, Juntao Feng, Wendong Xu

**Affiliations:** ^1^ Department of Hand Surgery Huashan Hospital Fudan University Shanghai China; ^2^ Department of Hand and Upper Extremity Surgery Jing’an District Central Hospital Fudan University Shanghai China; ^3^ The National Clinical Research Center for Aging and Medicine Huashan Hospital Fudan University Shanghai China

**Keywords:** central plasticity, contralateral c7 transfer, brachial plexus injury, synchronous sensation, total brachial plexus avulsion injury

## Abstract

**Backgrounds:**

Contralateral cervical seventh (C7) nerve transfer aids motor and sensory recovery in total brachial plexus avulsion injuries (TBPI), but synchronous sensation often persists postoperatively. The mechanism underlying synchronous sensory phenomena remain largely unknown.

**Objective:**

To investigate the role of central plasticity in sensory recovery after contralateral C7 nerve transfer.

**Methods:**

Sixteen right TBPI patients who received contralateral C7 nerve transfer for more than 2 years were included. Sensory evaluations included Semmes–Weinstein monofilament assessment (SWM), synchronous sensation test, and sensory evoked action potential (SNAP) test. Smaller value in the SWM assessment and larger amplitude of SNAP indicates better tactile sensory. Functional magnetic resonance imaging was performed while stimulations delivered to each hand separately in block‐design trials for central plasticity analysis.

**Results:**

The SWM value of the injured right hand was increased compared with the healthy left side (difference: 1.76, 95% confidence interval: 1.37–2.15, *p* < .001), and all 16 patients developed synchronous sensation. In functional magnetic resonance imaging analysis, sensory representative areas of the injured right hand were located in its ipsilateral S1, and 23.4% of this area overlapped with the representative area of the left hand. The ratio of overlap for each patient was significantly correlated with SWM value and SNAP amplitude of the right hand.

**Conclusion:**

The tactile sensory functioning of the injured hand was dominated by its ipsilateral SI in long‐term observation, and its representative area largely overlapped with the representative area of the intact hand, which possibly reflected a key mechanism of synchronous sensation in patients with TBPI after contralateral C7 transfer.

## Graphical abstract

The tactile sensory of the injured hand was dominated by its ipsilateral SI in long‐term observation, and its representative area overlapped largely with representative area of the intact hands, which was the key mechanism for synchronous sensory phenomenon in patients with TBPI after contralateral C7 transfer.

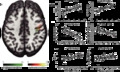

## 1 INTRODUCTION

Among injuries to the nerves of the upper extremity, total brachial plexus avulsion injury (TBPI) is one of the most devastating trauma, leading to almost complete loss of the sensory and motor function of the whole upper limb (Wade et al., 2019). To recover motor function of the paralyzed hand, the contralateral cervical seventh (C7) nerve, which contains both motor and sensory fibers, was transferred to the median nerve of the paralyzed hand (Y. D. Gu et al., 1992). This surgery is widely used for treating brachial plexus injuries, and has recently been adapted to treat hemiplegia after chronic cerebral injury (Waikakul et al., 1999; Zheng et al., 2018).

From a neuroanatomical perspective, this surgery connects the injured hand to the ipsilateral hemisphere through peripheral nerve rewiring. Therefore, it is crucial to determine whether patients have acquired independent motor and sensory function. Previous studies reported that, after approximately 2 years’ of remodeling and adaptation, the injured hand can acquire independent motor function and recover original levels of motor control function via the contralateral motor cortex(Y. Gu et al., 2002; Hua et al., 2013). However, synchronous sensory phenomena often persist, in which touching the injured hand can induce a sensation of tingling in the health hand (Chen et al., 2007; Y. Gu et al., 2002). Rather than improving the sensory integration of bilateral hands, synchronous sensation can induce misperception of the injured hand. As sensory‐motor integration is fundamental for motor reconstruction, effective sensory feedback is indispensable for motor learning (Wolpert & Flanagan, 2010), and restoring the sensory function of the paralyzed hand is important for the recovery of the injured hand (Bolognini et al., 2016). Therefore, understanding the mechanism underlying synchronous sensation is important for improving treatment. Previous animal research has indicated that central plasticity may play an important role in this phenomenon, by showing that the sensory perception of the injured forelimb is restricted in its ipsilateral hemisphere (Wang et al., 2010). However, the relationship between synchronous sensation and central plasticity remains unclear, and further clinical evidence is required to evaluate the weight of central plasticity for synchronous sensation after contralateral C7 nerve transfer.

Block‐design functional magnetic resonance imaging (fMRI) analysis can reveal the brain plasticity patterns, and has been widely used for detecting intervention‐related brain activity changes in disease models. In the current study, we applied block‐design fMRI scanning to explore sensory stimulus‐induced brain activity pattern in long‐term contralateral C7 nerve transfer patients, and its relationship with the recovery of sensory function in the paralyzed hand of patients with TBPI.

## 2 METHODS

### 2.1 Participants

Patients with TBPI who received contralateral C7 nerve transfer were recruited for this study. The following inclusion criteria were used:

1. TBPI proven by history of traumatic injury, loss of denervation of the injured arm in electrophysiological testing, and recordings of C5‐T1 nerve root rupture in surgical exploration.
2. The surgery strategy was as follows: the contralateral C7 nerve was transferred to the median nerve of the paralyzed hand, while the ulnar nerve was grafted and used to bridge in the C7 nerve transfer.
3. Injury on the right side, age over 18 years old (adult), with no restriction on sex.
4. The interval between surgery and fMRI scannings was more than 2 years.
5. This study was approved by the IRB of Huashan Hospital, and informed consent was acquired from all patients included in the study.

### 2.2 Sensory evaluations

As the pulp of the index finger of the injured hand was the most typical site for inducing synchronous sensation, evaluations were performed at this site. Three tests were used, including tactile threshold assessment, synchronous sensation test, and sensory electrophysiological testing.

To assess tactile threshold, a set of Semmes–Weinstein monofilaments (SWM, Bioseb, Vitrolles, France) were used. There are 20 levels of SWM, classified by the force (in grams) required to bend the monofilament perpendicularly against the skin. The values of the SWM assessments were expressed in log (10 × F; with *F* = force in milligrams), 1.90 to 6.48. The smallest unit detected by each participant in three out of five tests was used as the tactile threshold. The uninjured side was tested, followed by the injured side.

Synchronous sensation testing was performed after the SWM assessment. A filament, two grades greater than the tactile threshold, was applied to the injured index finger pulp to induce synchronous sensation. The synchronous sensation was classified into three levels: obvious, slight, or none. The test was repeated for three times in each patient.

For sensory electrophysiological testing, stimulation was delivered to the median nerve percutaneously at a distance of 2cm distal to the rasceta. A circular surface recording electrode was placed at the index finger. The latency and amplitude of the sensory nerve action potential (SNAP) were recorded for all patients three times, and the average value was used. The uninjured side was tested first, followed by the injured side.

### 2.3 Functional magnetic resonance imaging (fMRI) data acquisition

Participants were placed supine in a 32‐channel head coil on a 3T GE MR750 scanner. A foam pillow and a band across the forehead were used to restrict head movements. The following modular were scanned: (A) Block‐design functional MRI: T2*‐weighted single‐shot echo planar imaging (EPI) sequence, repetition time (TR) = 3,000 ms, echo time (TE) = 30 ms, field of view (FOV) = 220 × 220 mm^2^, slice number = 43, slice thickness = 3.2 mm (voxel size 3.4 × 3.4 × 3.2 mm^3^) matrix = 64 × 64, flip angle = 90°, and number of acquisitions = 60 (B)Structural MRI: 3D T1‐weighted SPGR sequence, sagittal slices = 180 with 2 slices in each end discarded to achieve 176, matrix size = 256 × 256, field of view = 256×256 mm^2^, repetition time (TR) = 8100 ms, echo time (TE) = 3.1 ms, flip angle (FA) = 8°, slice thickness = 1 mm, and voxel size = 1×1 × 1 mm^3^.

For the block‐design fMRI, two trials were performed for each patient. Each trial contained a paradigm of 30‐s mechanical stimulation followed by a 30‐s rest interval, repeated three times. The first trial involved mechanical stimulation of the index finger of the injured right hand, and the second trial involved stimulation of the index finger of the intact left hand. Prior to the first stimulation block, there was a 12‐s prescan period to obtain a stable baseline blood‐oxygen‐level‐dependent signal, and the prescan data were excluded from analysis.

### 2.4 fMRI data analysis

The imaging data underwent preprocessing and postprocessing steps using SPM12 (Statistical Parametric Mapping, University College of London, UK). The first four prescan volumes were removed for each participant, and slice timing correction was performed on the remaining images. The images were then registered to each subject's structural MRI data (six‐parameter rigid body, sinc interpolation; second order adjustment for movement). Anatomical T1 images were used for segmentation, then subjected to the normalization and smoothing (6 mm^3^ full width at half maximum) procedure.

Individual subject‐level statistical analyses were performed using the general linear model in SPM12. One active condition and one rest condition (baseline) were modeled using a canonical hemodynamic response function. A contrast map of active versus rest was obtained.

In the group level, one‐sample *t* test was used to detect the average activation area by task. The task data were divided into two subgroups: right hand stimulation (A), and left hand stimulation (B). Paired two‐sample *t* tests were used for comparison of A and B. The rate of overlap of activated brain areas was calculated in each patient and in the group level using the following formula:

$$\text{Overlapping rate}=\frac{A\cap B}{A\cup B}$$

### 2.5 Statistical analysis

For the unmatched analysis, descriptive statistics were used to report the characteristics of patients at baseline. Paired *t* tests were used for between‐group comparisons. In subgroup analysis, student's *t* tests were used for between‐group comparisons. To evaluate the weight of brain functional plasticity in incomplete sensory recovery of patients with TBPI, Pearson's correlation was used to analyze the relationship between sensory evaluation and brain plasticity index results, and the rate of overlap of each patient. Sensory evaluations included original values of SWM assessments, SNAP amplitude of the right hand, and the normalized value (value of the right hand divided by the value of the left hand in each patient (software: SPSS 22.0, IBM Tech.).

In fMRI analysis, voxels were considered to be significantly activated if they survived false discovery rate (FDR) correction (*q* < .05) for single subject analyses. We also performed a group analysis for each session across subjects using a one‐sample *t* test, with an FDR‐corrected threshold of *p* < .05.

## 3 RESULTS

### 3.1 Subjects

Thirteen male and three female right TBPI patients were enrolled in this study. The mean age was 26.7 ± 5.4 years, and the mean interval between contralateral C7 nerve transfer surgery and fMRI scanning was 4.2 ± 0.9 years. Demographic data and functional evaluation data were presented in Table 1.

**TABLE 1:** Demographic information and sensory test results of the patients

Case No.	Sex	Age (years)	Injury side	Mechanism of injury	Interval^a^ (years)	SWM^b^ (log)		SNAP^c^				Synchronous Sensory^d^
								Right		Left		
						Right	Left	Amplitude(μV)	Latency (ms)	Amplitude(μV)	Latency (ms)	
1	M	36	R	Automobile	5.4	4.60	2.30	15	9.5	41	2.6	Obvious
2	M	21	R	Motorcycle	3.1	3.78	2.60	20	7.3	56	2.7	Little
3	F	19	R	Fall	6.0	4.15	2.30	16	8.6	47	3.1	Obvious
4	M	25	R	Motorcycle	4.0	3.78	2.85	22	9.3	53	2.8	Little
5	M	21	R	Motorcycle	4.2	4.78	2.60	12	10.1	51	3.4	Little
6	M	23	R	Motorcycle	3.8	2.60	2.60	21	4.9	42	3.7	Little
7	M	33	R	Automobile	3.0	4.00	2.60	18	9.7	45	3.0	Obvious
8	M	28	R	Motorcycle	4.3	4.15	2.30	12	9.8	41	3.2	Little
9	M	34	R	Motorcycle	3.8	3.78	1.90	9	10.6	48	3.5	Obvious
10	F	26	R	Fall	4.6	5.18	2.85	14	10.5	37	3.5	Obvious
11	M	31	R	Fall	5.6	4.00	2.60	16	8.4	41	2.9	Obvious
12	M	27	R	Motorcycle	3.7	4.00	2.30	17	9.2	40	2.6	Obvious
13	M	20	R	Motorcycle	3.0	4.15	2.60	19	11.3	55	2.8	Obvious
14	F	33	R	Fall	4.1	4.00	2.30	21	7.6	45	3.4	Little
15	M	25	R	Automobile	3.7	5.00	2.30	9	9.3	42	2.9	Obvious
16	M	25	R	Motorcycle	5.1	5.78	2.60	7	11.7	36	3.5	Obvious

Note

M: male; F: female. R: Right.

^a^ Interval: The time between contralateral C7 nerve transfer surgery and functional MRI scanning.

^b^ SWM: The Semmes–Weinstein monofilament assessment, the results referred to the minimal necessary force in grams in bending the filament to induce tactile sensation at the index finger pulp, as expressed in log(10 × F; with *F* = force in milligrams), 1.90 to 6.48.

^c^ SNAP: Sensory nerve action potential.

^d^ Synchronous sensory: The extent of synchronous sensory of the donor side (left hand) accompanying regained function of the repaired nerve of the injured side (right hand).

### 3.2 Sensory evaluations

Compared with the uninjured left hand (mean: 2.48, 95%confidence interval [CI]: 2.34–2.60), the tactile threshold of the injured right hand by SWM assessment was significantly higher (mean: 4.23, 95%CI: 3.85–4.62), indicating incomplete sensory recovery, as shown in Table 1.

All 16 patients developed synchronous sensation, which was classified as “obvious” in 10 patients and “little” in 6 patients. Subgroup analysis based on the extent of synchronous sensation showed that patients with obvious synchronous sensation exhibited a significantly higher rate of overlap. The tactile threshold of SWM assessments of the obvious subgroup was higher than that of slight group (difference: 0.62, 95%CI: −0.13 to 1.4, *p* = .100), although the difference was not statistically significant (Table 2).

**TABLE 2:** Subgroup analysis on synchronous sensory of sensory tests of the injured hand

	Obvious^a^—Mean(*SD*)	Little^a^—Mean(*SD*)	Difference—Mean(95%CI)	*p* value
Number of patients	10	6	4	‐
Age—years	27.6 (5.8)	25.1 (4.7)	2.4(−3.6 to 8.4)	.398
Interval—years	4.39 (1.10)	3.92 (0.44)	0.47(−0.54 to 0.47)	.334
SWM^b^ (log)	4.46 (0.66)	3.85 (0.71)	0.62(−0.13 to 1.4)	.100
Amplitude of SNAP^c^—μV	14.0 (4.2)	18.0 (4.7)	−4.0(−8.8 to 0.8)	.098
Latency of SNAP—ms	9.9 (1.1)	8.2 (2.0)	1.7 (0.1 to 3.3)	.041
Overlapping rate—%	30.0 (9.6)	12.3 (4.2)	15.7 (8.7 to 26.6)	<.001

^a^ “Obvious” and “Little” refers to the patient report of synchronous sensory test.

^b^ SWM: The Semmes–Weinstein monofilament assessment, the results referred to the minimal necessary force in grams in bending the filament to induce tactile sensation at the index finger pulp, as expressed in log(10 × F; with *F* = force in milligrams), 1.90 to 6.48.

^c^ SNAP: Sensory nerve action potential.

Sensory neurophysiological tests showed that the mean amplitude of SNAP of the injured right hand was 15.5 ± 4.7 μV, compared with 45.0 ± 6.2μV for the uninjured left hand (difference: −29.5, 95%CI: −32.6 to −26.4, *p* < .001). The mean latency of SNAP of the injured right hand was 9.2 ± 1.7 ms, compared with 3.1 ± 0.4 ms for the uninjured hand (difference: 6.1, 95%CI: 5.2 to 7.1, *p* < .001).

### 3.3 fMRI analysis

**FIGURE 1:**
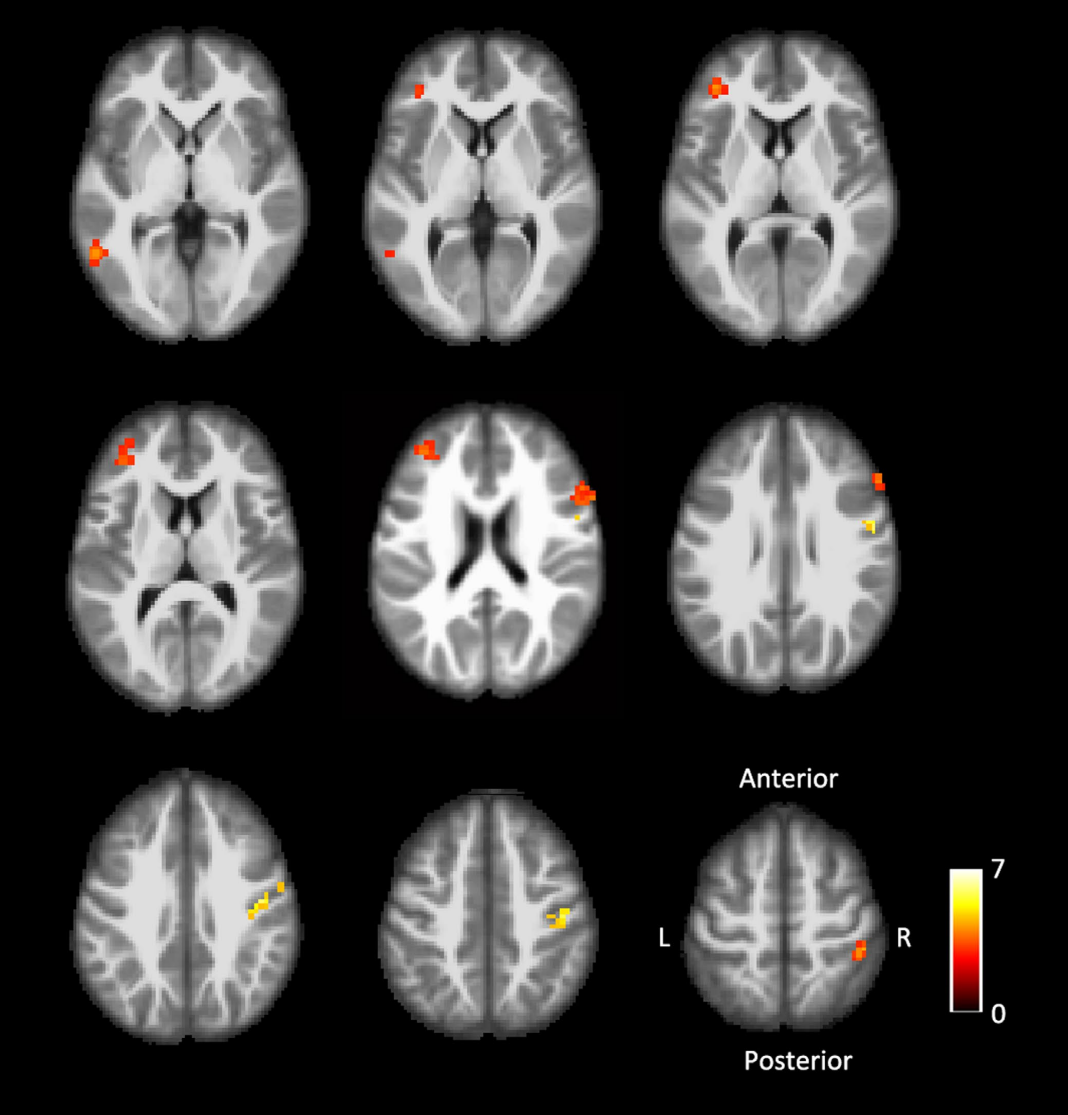
Functional MRI assessments of the injured (right) hand stimulation. Images showing different brain slices in an axial surface. In each image, t values (a statistic indicating the strength of brain activation in each voxel) in the analyses comparing mechanical stimulation of the injured hand with resting are indicated on a color scale (color intensity ranges from 0 to 7, with higher values indicating higher t values and stronger activation in a given voxel)

**FIGURE 2:**
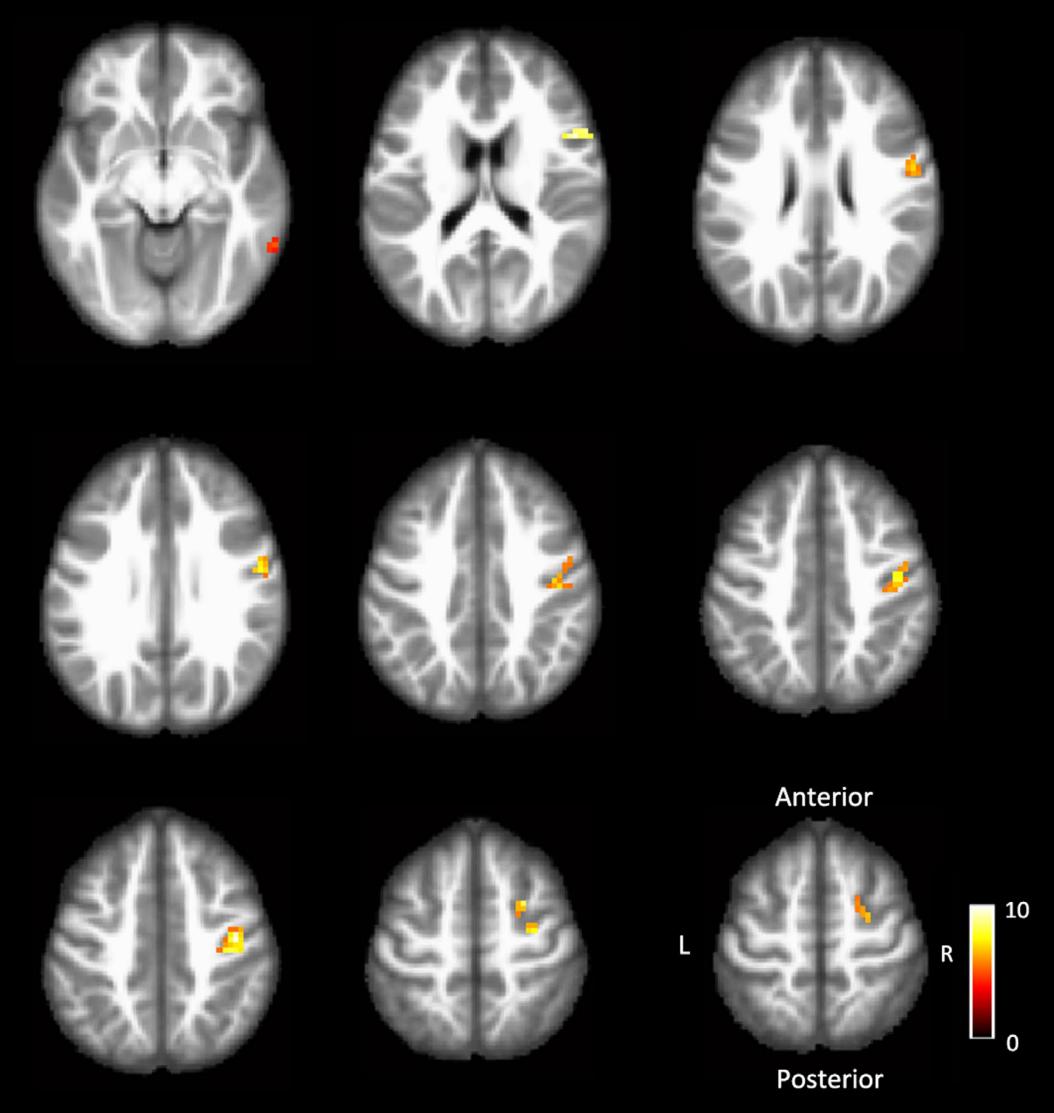
Functional MRI assessments of the intact (left) hand stimulation. Images showing different brain slices in an axial surface. In each image, t values in the analyses comparing mechanical stimulation of the intact hand with resting are indicated on a color scale (color intensity ranges from 0 to10, with higher values indicating higher t values and stronger activation in a given voxel)

**FIGURE 3:**
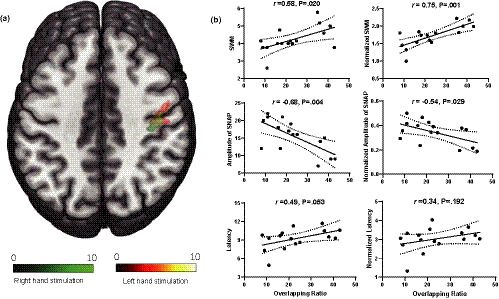
The relationship between activation area in SI and sensory evaluations of bilateral hands. Panel A showed group level analysis in overlapping of brain activation areas by stimulation of right (green) and left (hot) hand in S1. Panel B showed the correlation analysis of different sensory evaluations and ratio of overlap in each patient. SWM: The Semmes–Weinstein monofilament assessments. SNAP: Sensory nerve action potential

In all 16 patients, right hand stimulation activated the ipsilateral postcentral cortex, without activation of its contralateral left postcentral cortex. In addition, other regions including right precentral gyrus, bilateral frontal gyrus, and left middle temporal gyrus showed activation (Figure 1, Table 3). Stimulation to the uninjured left hand activated its contralateral right postcentral gyrus, as well as right precentral gyrus, right frontal gyrus, and right middle temporal gyrus (Figure 2, Table 3). In the group analysis, the rate of overlap of the activated area in S1 by stimulation to the right and left hand stimulation was 23.4%, as shown in Figure 3.

**TABLE 3:** Group analysis of the index finger stimulation activated brain areas in the patients

Stimulation site	Brain regions	Number of voxels	Peak coordinates(mm)^a^			Peak *t* value^b^
			x	y	z	
Right index finger	Right postcentral gyrus	67	54	−6	33	7.33
	Right precentral gyrus	23	45	−18	48	6.08
	Left inferior frontal gyrus	96	−39	42	6	4.27
	Right inferior frontal gyrus	43	57	24	27	4.27
	Left middle temporal gyrus	24	−57	−57	1	4.25
Left index finger	Right postcentral gyrus	83	54	−6	30	10.58
	Right precentral gyrus	33	42	−15	48	8.15
	Right superior frontal gyrus	21	27	3	54	8.54
	Right inferior frontal gyrus	38	51	12	18	9.92
	Right middle temporal gyrus	22	63	−51	−9	5.06

^a^ Peak coordinates: The coordinates of the voxel with peak value of each cluster in the MNI coordinate space (MNI = Montreal Neurological Institute).

^b^ Peak t value refers to the t value of the peak point.

### 3.4 Correlation analysis

We further analyzed the rate of overlap rate of each patient and its relationship with sensory evaluation results. The tactile threshold by SWM assessments, amplitude of SNAP, and their normalized values were significantly correlated with the overlap ratio (Figure 3). The results revealed no significant correlation between overlap ratio and latency (*r* = .49, *p* = .053), or between overlap ratio and normalized latency (*r* = .34, *p* = .192).

## 4 DISCUSSION

In the current study, we evaluated the relationship between brain plasticity and sensory recovery, and a common synchronous sensory phenomenon in patients with TBPI after contralateral C7 nerve transfer surgery. As this surgery involves rewiring of one of the five nerve roots from the intact side to the injured side, it connects the injured hand to its ipsilateral SI. The current results showed that, unlike the classical cross‐dominance model, the right SI can begin to serve as the sensory center for its ipsilateral hand (the injured right hand) in long‐term follow‐up. Moreover, the representative area of the injured right hand largely overlapped with the representative area of the intact left hand, and the overlap ratio was significantly correlated with the sensory recovery, potentially reflecting the central mechanism underlying synchronous sensory phenomena.

A previous study reported an “interhemispheric” remodeling pattern of M1 in patients with TBPI after contralateral C7 nerve transfer (Hua, Li, et al., 2012; Hua, Zuo, et al., 2012). In a follow‐up period of 3 years or longer, the silent contralateral M1 was found to be reactivated by movement of the injured hand (Beaulieu et al., 2006; Liu et al., 2013). In addition, the injured hand was reported to regain independent movement function(Y. Gu et al., 2002). It should also be noted that before independent movement recovery, motor function also undergoes a period of synchronous motor activity, mostly taking place between 2 and 5 years after surgery (Beaulieu et al., 2006; Hua, Zuo, et al., 2012; Lanaras et al., 2009; Waikakul et al., 1999). Therefore, in the current study, most patients reached or even exceeded the synchronous period observed in motor remodeling, but the sensory perception of the injured hand could still not be separated from the intact hand.

Understanding synchronous sensory phenomenon is important, not only because it is a unique neuroscientific feature, but also because it may help to find a way to promote motor recovery for surgeons and therapists. Sensory feedback is important for the motor relearning process, particularly in peripheral nerve injuries and prosthesis training for amputees (Hattori et al., 2009; Yao et al., 2015). Through functional and structural integration of the sensory and motor cortices, motor control can be enhanced, improving motor recovery (Ostry & Gribble, 2016; Singh & Scott, 2003).

Two factors can potentially hinder this process: incomplete peripheral nerve axonal loss and aberrant sensory related central plasticity. Because sensory electrophysiological tests and tactile evaluations have been demonstrated to promote the successful regeneration of the sensory nerve, peripheral nerve regeneration is unlikely to be the main cause of synchronous sensation. Rather, because the overlapping ratio is significantly related to the incomplete sensory recovery, aberrant central plasticity is more likely to be the main cause of this phenomenon (Wang et al., 2010; Zuo et al., 2010). This type of erroneous perception could potentially influence motor recovery of the injured hand by impeding normal sensory feedback circuits.

Two major issues should be investigated in future studies of TBPI and central plasticity: how to separate the overlapping areas of the two hands and how to reactivate the silent contralateral S1 of the injured hand. Recent progress in mirror movement therapy, repetitive transcranial magnetic stimulation therapy, and multidimensional motor learning exercises strongly indicate the importance of central intervention in the rehabilitation of central and peripheral nerve injury rehabilitation (Borich et al., 2015; Chaudhary et al., 2016; Cramer et al., 2011). The current findings suggest that separating representations of the injured hand may be important for tactile sensory recovery. Therefore, central interventional methods including sensory transcranial magnetic stimulation, visual‐sensory feedback rehabilitation, and tactile enhancement methods could be used to further promote sensory recovery. More attentions should be paid to the sensory cortex in future research.

## 5 CONCLUSION

Tactile sensation of the injured hand was dominated by its ipsilateral SI in long‐term observation, and its representative area largely overlapped with the representative area of the intact hand, possibly reflecting the key mechanism underlying for synchronous sensory phenomena in patients with TBPI after contralateral C7 transfer.

## 6 Ethic committee approval

The study was approved by the institutional review board of Huashan Hospital and registered on www.chictr.org.cn (number: ChiCTR‐IIR‐13004466).

## 7 DATA AVAILABLE STATEMENT

The data that support the findings of this study are available from the corresponding author upon reasonable request.

## CONFLICT OF INTEREST

None declared.

## AUTHOR CONTRIBUTION

WD Xu, YD Gu, and JT Feng designed the study. ZY Cai and GW Lei collected and prepared the data. JT Feng and ZY Cai analyzed and interpreted the data. JT Feng, ZY Cai, J Li and YD Shen drafted the article. All of the authors critically revised the article. JT Feng approved the final version of the manuscript on behalf of all authors. WD Xu supervised the study.
